# Prevalence, Molecular Characteristics and Virulence Identification of Bovine Parainfluenza Virus Type 3 in China

**DOI:** 10.3390/v16030402

**Published:** 2024-03-05

**Authors:** Xiaowen Xu, Wanyue Zhao, Zhijie Xiang, Chen Wang, Mingpu Qi, Sen Zhang, Yuanchen Geng, Yuhao Zhao, Kaihui Yang, Yanan Zhang, Aizhen Guo, Yingyu Chen

**Affiliations:** 1 National Key Laboratory of Agricultural Microbiology, College of Veterinary Medicine, Huazhong Agricultural University, Wuhan 430070, China; xiaowenxu@webmail.hzau.edu.cn (X.X.); wanyuezz@163.com (W.Z.); xiangzhijie2024@163.com (Z.X.); wangchen2012@webmail.hzau.edu.cn (C.W.); qimingpu@126.com (M.Q.); zhangs416@webmail.hzau.edu.cn (S.Z.); yuanchen@webmail.hzau.edu.cn (Y.G.); zhaoyuhao@webmail.hzau.edu.cn (Y.Z.); yangkaihui@webmail.hzau.edu.cn (K.Y.); zyn3203349246@163.com (Y.Z.); 2 Hubei International Scientific and Technological Cooperation Base of Veterinary Epidemiology, The Cooperative Innovation Center for Sustainable Pig Production, Huazhong Agricultural University, Wuhan 430070, China; 3 Key Laboratory of Development of Veterinary Diagnostic Products, Huazhong Agricultural University, Ministry of Agriculture and Rural Affairs, Wuhan 430070, China; 4 Hubei Hongshan Laboratory, Huazhong Agricultural University, Wuhan 430070, China

**Keywords:** bovine parainfluenza virus type 3, isolation and identification, genetic characteristic, pathogenicity

## Abstract

Bovine parainfluenza virus type 3 (BPIV-3) is one of the major pathogens of the bovine respiratory disease complex (BRDC). BPIV-3 surveillance in China has been quite limited. In this study, we used PCR to test 302 cattle in China, and found that the positive rate was 4.64% and the herd-level positive rate was 13.16%. Six BPIV-3C strains were isolated and confirmed by electron microscopy, and their titers were determined. Three were sequenced by next-generation sequencing (NGS). Phylogenetic analyses showed that all isolates were most closely related to strain NX49 from Ningxia; the genetic diversity of genotype C strains was lower than strains of genotypes A and B; the HN, P, and N genes were more suitable for genotyping and evolutionary analyses of BPIV-3. Protein variation analyses showed that all isolates had mutations at amino acid sites in the proteins HN, M, F, and L. Genetic recombination analyses provided evidence for homologous recombination of BPIV-3 of bovine origin. The virulence experiment indicated that strain Hubei-03 had the highest pathogenicity and could be used as a vaccine candidate. These findings apply an important basis for the precise control of BPIV-3 in China.

## 1. Introduction

Bovine respiratory disease complex (BRDC), commonly called “shipping fever”, is a multi-factor disease resulting from the combined actions of environmental and microorganisms. It leads to substantial economic losses because of a 60–90% morbidity in feedlots [1,2,3]. Bovine parainfluenza virus type 3 (BPIV-3), a member of the Respirovirus bovis in the Paramyxoviridae family, is one of the most essential viruses of BRDC. It usually damages the respiratory tissues, triggers interstitial pneumonia in cattle, leads to immunosuppression [4], and heightens susceptibility to secondary bacterial infections, leading to more severe clinical signs [5].

BPIV-3 was first isolated and identified in BRDC cases in 1959 [6]. It is a single-segmented, single-stranded, negative-sense RNA virus [7,8] encoding six structural proteins (N, P, L, M, HN, and F) and three non-structural proteins (C, V, and D) [9,10]. Currently, BPIV-3 is divided into three genotypes: A, B, and C [11]. Genotype B was classified primarily according to phylogenetic analysis of the nucleotide sequences for the M gene and the whole genome, whereas the classification of genotype C was based on phylogenetic analysis of the nucleotide sequences for HN gene and the entire genome [12,13]. All three genotypes have been reported in China, and the prevalent predominant genotypes vary by place and time [4,8,14]. With the rapid development of the cattle industry in China, BPIV-3 spread rapidly in Northern Chinese pastures, with antibody positivity reaching as high as 80% [15]. However, data on the pathogen epidemiology and genetic characterization of BPIV-3 are still very limited, which brings a great challenge to the prevention and control of BPIV-3, even BRDC.

To understand the epidemiology and genomic characteristics of BPIV-3, we systematically investigated its prevalence of BPIV-3 in China and analyzed its molecular features. In the current study, samples of 302 cattle were collected from cattle farms of eight provinces and two autonomous regions in China where BRDC outbreaks have occurred. Six strains of BPIV-3 were obtained from 14 PCR-positive nasal swab samples. Three were sequenced, and the phylogenetic analyses virulence was then assessed using guinea pig models. The findings contribute to a better knowledge of the evolution and molecular characteristics of BPIV-3 in China, and they can be employed in developing a vaccine candidate for BPIV-3 control.

## 2. Materials and Methods

### 2.1. Samples and Treatment

In September 2021 and July 2023, a total of 302 cattle samples (including 281 nasal swabs and 21 lung tissues) were collected from cattle farms of eight provinces and two autonomous regions in China where BRDC outbreaks have occurred (Supplementary Table S1).

Samples (tissue was first homogenized and centrifuged at 8000 rounds (r)/min for 3 min) were frozen and melted several times. RNA extraction and cDNA synthesis were conducted according to the manufacturer’s instructions. DNA/RNA Extraction Kit and HiScript^®^ II 1st Strand cDNA Synthesis Kit were from Vazyme Biotech Co., Ltd. (Nanjing, China).

### 2.2. PCR

The primers and probes specifically designed for this study are provided in Supplementary Table S2. Primer 1 was used to detect BPIV-3 in cattle samples. Primers 2 and 3 were used to identify the genotype. Primer 2 presented genotype A, and Primer 3 presented genotype C. The reaction conditions were as follows: 98 °C for 2 min; 95 °C for 10 s, annealing at (Primer 1: 57.5 °C, Primer 2: 56 °C, Primer 3: 55 °C) for 15 s, 72 °C for 15 s, 35 cycles; 72 °C for 5 min. Positive PCR products were sent to Beijing Tsingke Biotech Co., Ltd. (Beijing, China) for sequencing.

### 2.3. Virus Isolation and Characterization

#### 2.3.1. Virus Isolation and Identification

Madin–Darby bovine kidney cells (MDBK) were purchased from the China Institute of Veterinary Drug Control (Beijing, China).

The BPIV-3-positive nasal swab samples (1.5 mL of DMEM was added at the time of collection) were frozen and melted several times, and the supernatants were collected and filtered through 0.22 μm filters. The filtered supernatants were inoculated into MDBK cells. The viruses were harvested 3–5 days post-infection or when over 80% of the cells showed cytopathic effects. The harvested solution underwent three freeze–thaw cycles, centrifuged at 4000 r/min for 10 min at 4 °C, and the supernatant was collected. After filtering through a 0.22 μm filter, it was stored at −80 °C for future use. PCR was used to identify and type cultures producing cytopathic effects.

#### 2.3.2. Transmission Electron Microscopy

The isolated viruses were purified through plaque purification to obtain pure strains. After concentration and purification viral particles were resuspended in PBS. A volume of 50 μL of virus resuspension was sent to Wuhan Servicebio Technology Co., Ltd. (Wuhan, China) for electron microscopy.

#### 2.3.3. The Viral Titer and One-Step Growth Curve

The viral titer was measured by median tissue culture infectious dose (TCID_50_) based on the Reed–Muench method.

The one-step growth curve was conducted as follows: MDBK cells that had reached confluence were inoculated with BPIV-3 isolates at 0.1 MOI (multiplicity of infection). Virus samples were collected at 3, 6, 9, 12, 18, 24, 30, 36, 42, 48, 60, and 72 h after inoculation. The obtained virus was frozen and thawed three times before the viral titer was evaluated using TCID_50_.

### 2.4. Whole-Genome Sequencing and Phylogenetic Analysis

The inactive BPIV-3 strains were sent to Guangdong Meige Gene Technology Co., Ltd. (Shenzhen, China) for whole-genome sequencing (WGS) using NGS technology.

Geneious Prime software (2023.0.3) was used to compare the nucleotide sequences of different strains and construct phylogenetic trees. The information on reference strains is shown in Supplementary Table S3.

To analyze the gene recombination of the isolates, the sequences of isolates and 40 BPIV-3 strains were subjected to whole-genome multiple sequence alignment analysis. Potential recombination events were predicted and analyzed using RDP, GENECONV, Chimaera, MaxChi, BootScan, SiScan, and 3Seq in RDP4 (v4.101). A recombination event was considered relatively credible only if detected as significant (*p* < 0.05) by at least three methods mentioned above. Simplot (3.5.1) was used for the visualization analysis of the recombination events.

To analyze the protein variations in BPIV-3, the amino acid sequences of proteins from isolates and all genotype C strains were aligned. The tertiary structures of the proteins from isolates were predicted using alphafold2 (https://colab.research.google.com/github/sokrypton/ColabFold/blob/main/AlphaFold2.ipynb) (accessed on 4 November 2023). The amino acid mutation sites in different proteins of isolates were annotated using pymol (2.3.0).

### 2.5. Pathogenicity Detection Using Guinea Pig Model

Experimental animals were purchased from the Experimental Animal Center of Three Gorges University (Yichang, China), totaling 35 SPF female guinea pigs weighing 350–400 g each, and were housed in a negative-pressure animal room.

All 35 guinea pigs were divided into four groups. Group 1–3 (ten guinea pigs in each group) were infected with 10^7^ TCID_50_/mL (200 μL per nostril) Hubei-01, Hubei-02, and Hubei-03 strains via intranasal administration, respectively. Group 4 (five guinea pigs) was the control group (200 μL DMEM culture medium per nostril).

#### 2.5.1. Clinical Symptom Observation, Anatomical Pathology Examination, and Histopathological Changes

The body temperature of all guinea pigs was measured and recorded daily from 1 day before infection until the end of the experiment. The guinea pigs’ mental state, activity level, and any respiratory-related symptoms were observed daily.

On 1, 3, 5, 7, and 15 days post-infection, two guinea pigs from Groups 1–3 and one from Group 4 were euthanized. Visible lesions in organs and tissues such as the heart, liver, spleen, lungs, and kidneys were observed. 

Lungs were immediately put in a 10% neutral buffered formalin solution for fixation and kept for histopathological examination. The results of pathological sections were analyzed and scored according to the international standards for rat and mouse pathological changes terminology and diagnostic criteria (Table 1).

#### 2.5.2. Detoxification Duration and Organ Viral Load

Nasal swabs obtained from guinea pigs on days 1, 3, 5, 7, 9, 11, 13 and 15 post-infection were cultured with cells to determine the cytopathic effect (CPE). Nucleic acids were extracted for qPCR.

The lungs were weighed to measure the organ viral load. An appropriate amount of lung tissue was homogenized and centrifuged at 8000 r/min for 3 min; 100 μL of the supernatant was taken, diluted in a 10-fold series, and pipetted into a 96-well plate. The viral titer was determined after 3–5 days using the Reed–Muench method. A volume of 200 μL of the supernatant was utilized to extract RNA and subsequently convert it into cDNA for quantitative PCR (qPCR) analysis.

qPCR was conducted as follows: 95 °C for 5 min, 95 °C for 10 s, 60 °C for 30 s, and 40 cycles; the fluorescence signal was acquired in this step. Primers and probes are listed in Supplementary Table S2.

#### 2.5.3. Virus Neutralization Test, Hemagglutination Test, and Hemagglutination Inhibition Test

Blood from guinea pigs was collected on days 1, 3, 5, 7, 9, 11, 13, and 15 days post-infection. The serum was centrifuged at 4 °C for 5000 r/min for 5 min and then inactivated at 56 °C for 30 min for subsequent testing. 

The serum was diluted with phosphate-buffered saline (PBS) at a 2-fold ratio, and 50 μL of diluted serum was mixed with an equal volume of virus solution (200 TCID_50_/0.1 mL) in a 96-well cell culture plate. The plate was then incubated at 37 °C for 1 h and added 100 μL of cells. Subsequently, the 96-well cell culture plate was incubated at 37 °C in a 5% CO_2_ incubator for 3–5 days. The CPE was observed, and the neutralization antibody titer was calculated using the Reed–Muench method.

A V-shaped 96-well microplate was taken, and 25 µL of PBS was added to each well of columns 2–12 (3 wells per column, i.e., 3 replicates). A volume of 50 µL of virus solution (already concentrated and purified) was added to column 1, and 25 µL from column 1 was pipetted to column 2. A two-fold dilution was then made by mixing well, and 25 µL of diluted solution was pipetted from column 2 to column 3, and so on, until a two-fold dilution was made to column 11, diluting the virus solution from 2^0^ to 2^−10^. Column 12 served as a blank control. Subsequently, 25 µL of 1% guinea pig red blood cells were added to the wells. The microplate was placed in a 37 °C incubator and left to stand for 30 min. Then, the result was observed and recorded in time, and the hemagglutination titer of the virus was determined. The HA titer was considered the highest virus dilution in which complete agglutination occurred.

Four-unit antigens (Four HA units per 25 µL) were prepared based on the hemagglutination titer of the virus. A V-shaped 96-well microplate was taken, and 25 µL of PBS was added to each well of columns 1–11 (3 wells per column, i.e., 3 replicates), while 50 µL of PBS was added to column 12. Then, 25 µL of serum to be examined was added to column 1 and mixed thoroughly. Subsequently, 25 µL of dilution was pipetted from column 2 to column 3, and so on, performing a 2-fold dilution until column 10. Column 11 served as the virus control, while column 12 was the erythrocyte control in the experimental setup. In columns 1 through 11, 25 µL of a 4-unit antigen was added, and the reaction plate was gently tapped to mix the reactants thoroughly. The microplate was then left to stand at room temperature for 30 min. Subsequently, 25 µL of 1% guinea pig erythrocytes was added from right to left, and the microplate was placed on a micro-volume oscillator for 1 min. Finally, the microplate was left to stand at room temperature for 40 min to observe the results. The HI titer was considered the highest dilution of serum in which complete agglutination did not occur.

#### 2.5.4. Specific Antibody Test

The specific antibody was tested using indirect ELISA. Briefly, ELISA plates were coated overnight at 4 °C with 1.2 μg/mL purified virus. After washing, block the plate with 2% fish gelatin sealing solution at 37 °C for 2 h. After washing, guinea pig serum samples were serially diluted and added, then incubated at 37 °C for 1 h. The enzyme-labeled anti-guinea pig monoclonal antibody was added to each well and incubated for 1 h at 37 °C before being washed. After properly cleaning, substrate solutions were applied for 10 min before stopping the process. The OD630 nm values were quickly obtained using an enzyme labeler.

### 2.6. Statistical Analysis

All statistical tests of the data were performed using GraphPad Prism software (version 8.3.0). The data are shown as mean ± SD of three independent or three replicate samples. The statistical significance was calculated using two-way ANOVA for multiple comparisons. *p* < 0.05 was considered to show statistically significant differences.

## 3. Results

### 3.1. Positive Rate of BPIV-3

In total, 14 of 302 cattle tested positive for PCR, with a 4.64% (95% CI: 2.56–7.66) (14/302) animal-level positive proportion and a 13.16% (95% CI: 4.41–28.09) (5/38) herd-level proportion.

### 3.2. Virus Isolation, Identification, Titer, and One-Step Growth Curve

**Figure 1:**
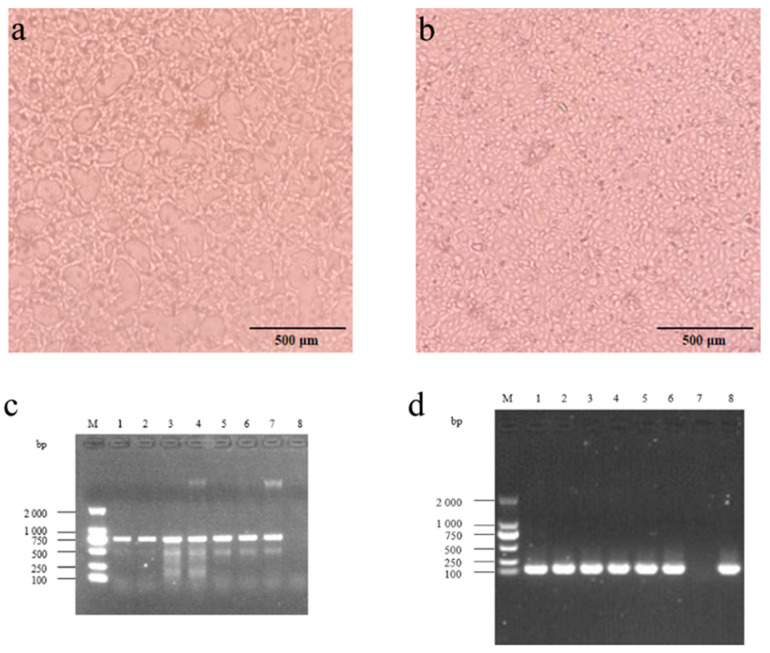
Virus isolation and identification. (**a**) MDBK cells after viral infection. (**b**) Control cells. (**c**) BPIV-3 PCR amplification results. M: DL2000; 1–6: BPIV-3 virus detection results from different samples; 8: negative control; 7: positive control. (**d**) BPIV-3C PCR amplification results. M: DL2000; 1–6: BPIV-3C virus detection results from different samples; 7: negative control; 8: positive control.

Virus isolation was then performed on all 14 PCR-positive nasal swab samples. Six obtained stable cell changes after 3–5 generations of cell inoculation (Figure 1a,b). PCR results identified that all six strains were BPIV-3C (Figure 1c,d). After plaque purification, we gained six purified BPIV-3C strains without other pathogen contamination. The six BPIV-3C strains were named BPIV-3C/Bovine/CHN/Hubei-01/2021 (abbreviated as BPIV-3C Hubei-01), BPIV-3C/Bovine/CHN/Hubei-02/2021 (abbreviated as BPIV-3C Hubei-02), BPIV-3C/Bovine/CHN/Hubei-03/2021 (abbreviated as BPIV-3C Hubei-03), BPIV-3C/Bovine/CHN/Hubei-04/2021 (abbreviated as BPIV-3C Hubei-04), BPIV-3C/Bovine/CHN/Hubei-05/2021 (abbreviated as BPIV-3C Hubei-05), and BPIV-3C/Bovine/CHN/Hubei-06/2021 (abbreviated as BPIV-3C Hubei-06), respectively. The six strains obtained above were isolated from three cattle farms in the cities of Xiantao, Huanggang, and Jingmen in Hubei Province, respectively.

**Figure 2:**
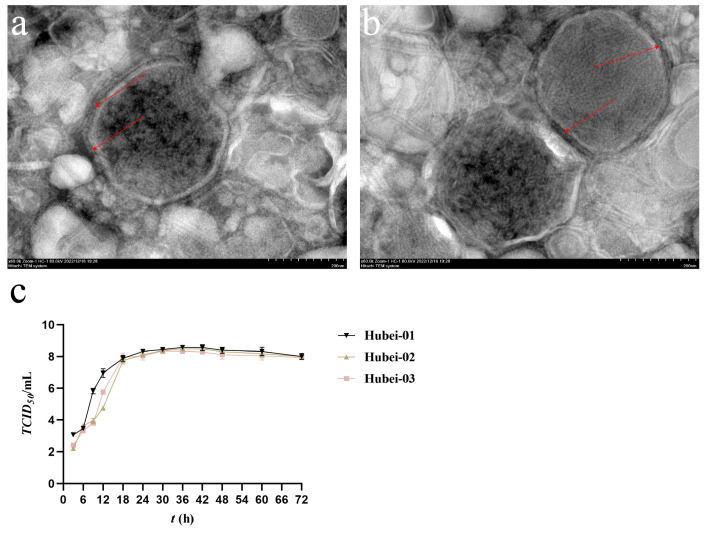
Characterization of BPIV-3. (**a**,**b**) are virus particles of BPIV-3, and numerous spikes were marked with red arrows. (**c**) Growth curve of BPIV-3C.

Under electron microscopic observation (Figure 2a,b), BPIV-3C virus particles were found to have a diameter between 200 and 250 nm. They were observed to be roughly spherical. A visible envelope enveloped the virus particle’s nucleocapsid, and numerous spikes were present on the envelope. The TCID_50_ of the six BPIV-3C strains was measured, with titers of 10^8.1^, 10^7.8^, 10^7.55^, 10^7.47^, 10^7.43^, and 10^7.2^ TCID_50_/mL, respectively.

As six strains were from three cattle farms, we chose one strain from each farm to detect the growth curves (Hubei-01, Hubei-02, and Hubei-03). Results showed that all strains replicated and increased rapidly within 0–18 h post-infection. Hubei-01 grew faster than the other two strains, followed by Hubei-03 and Hubei-02. At 36 h post-infection, all three strains reached their peaks, with virus titers as high as 10^8.57^, 10^8.48^, and 10^8.33^ TCID_50_/mL, respectively. They reached the plateau phase at 36–42 h post-infection and then decreased (Figure 2c).

### 3.3. Genome and Phylogenetic Analysis

#### 3.3.1. Identity and Phylogenetic Analysis

The complete genome lengths of BPIV-3C Hubei-01, Hubei-02, and Hubei-03 strains were 15,465 bp, 15,460 bp, and 15,462 bp, respectively (Supplementary Table S4).

**Figure 3:**
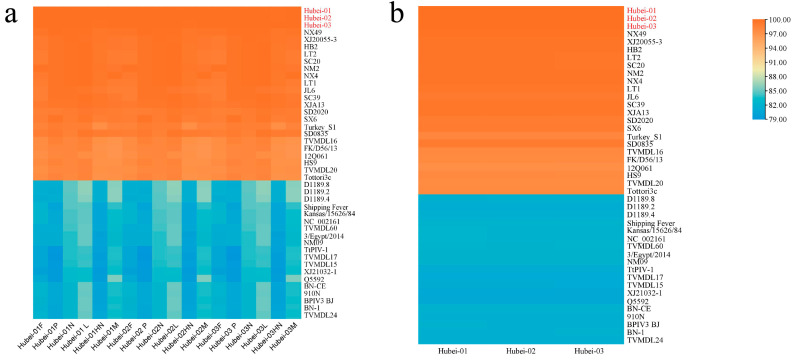
Identity analysis of BPIV-3. (**a**) Identity of nucleotide sequences in each gene region of BPIV-3 strain. (**b**) Identity of whole-genome nucleotide sequences of BPIV-3 strains. The Clustal Omega program in Geneious Prime was used to compare the identity between the 40 BPIV-3 strains published by Genbank and the isolates in each gene region, with the isolates represented by the red font.

In each gene region, Hubei-01, Hubei-02, and Hubei-03 were observed to have a high degree of identity with C genotype strains reported previously (Figure 3a), based on the identity analysis of the complete genome nucleotide sequences (Figure 3b), Hubei-01, Hubei-02, and Hubei-03 isolates had identity of 99.02–99.70%, 99.01–99.70%, and 99.00–99.68% with BPIV-3C strains reported in China, respectively, with the highest identity of 99.70%, 99.70%, 99.68% with the NX49 strain from Ningxia. Among the other countries, the isolates had the highest identity with the Turkish S1 strain of 98.171%, 98.170%, and 98.157%, respectively.

**Figure 4:**
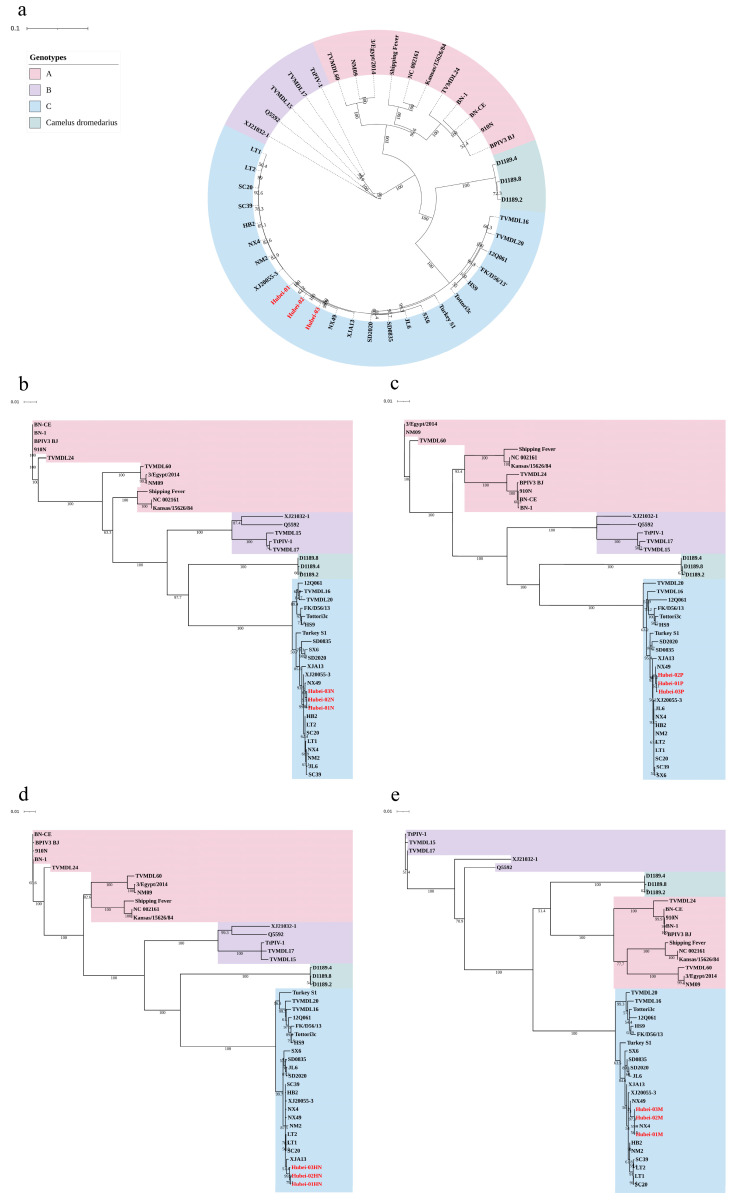
The phylogenetic trees were constructed based on the BPIV-3 nucleotide sequences. Using nucleotide sequences retrieved from 40 BPIV-3 strains in GenBank and 3 isolated strains, phylogenetic trees for BPIV-3 whole genome, HN, P, and N, were established. Isolates from this study were denoted in red font. (**a**) Whole-genome phylogenetic tree. (**b**) N gene phylogenetic tree. (**c**) P gene phylogenetic tree. (**d**) HN gene phylogenetic tree. (**e**) M gene phylogenetic tree. The evolutionary trees were constructed using the neighbor-joining method and tested with the bootstrap method. The numbers on the branches represent the percentage of 1000 bootstrap replications supporting each phylogenetic branch.

Based on the phylogenetic analysis of the complete genome nucleotide sequences, BPIV-3C Hubei-01, Hubei-02, and Hubei-03 exhibited a strong clustering pattern with other BPIV-3C strains, with a relatively low differentiation. All the isolated strains were genetically closest to the NX49 strain. Genotypes A and B, especially A, had higher differentiation. The strains sourced from three dromedary camels without typing were set on a separate branch and demonstrated to have a genetic distance closer to the C genotype (Figure 4a).

Further, phylogenetic trees were constructed based on different proteins. The typing results and phylogenetic relationships of HN, P, and N proteins aligned with the whole genome, but the M protein presented slight variations. The phylogenetic tree derived from the M protein indicated that the three BPIV-3 strains sourced from dromedary camels and the A genotype strains were grouped on the same branch, a pattern distinctly different from the evolutionary trees of the other proteins and the entire genome (Figure 4b–e).

#### 3.3.2. Recombination Analysis

**Figure 5:**
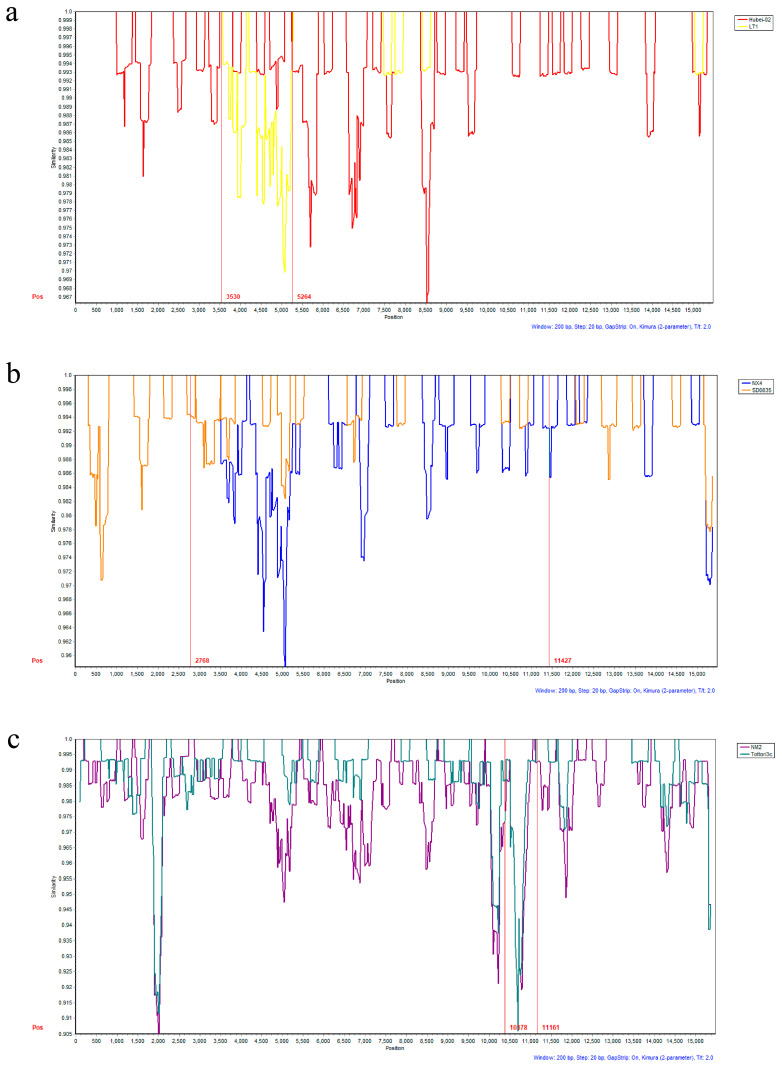
Simplot program to analyze genomic recombination events of BPIV-3 strains. (**a**) Recombination region of NX4 strain. (**b**) Recombination region of JL6 strain. (**c**) Recombination region of 12Q061 strain.

No recombination signals were detected in any of the strains isolated for this study. According to the database, C genotype strains like NX4, JL6, and 12Q061 exhibited one potential recombination event (Supplementary Table S5). Hubei-02 isolates from this study were identified as one of the likely parental strains for the recombination event in the NX4 strain. Subsequently, the recombination events of NX4, JL6, and 12Q061 were visually analyzed using Simplot, and the outcomes were consistent with the predictions from RDP4 (Figure 5).

#### 3.3.3. Amino Acid Variation in the Protein of BPIV-3 Isolates

**Figure 6:**
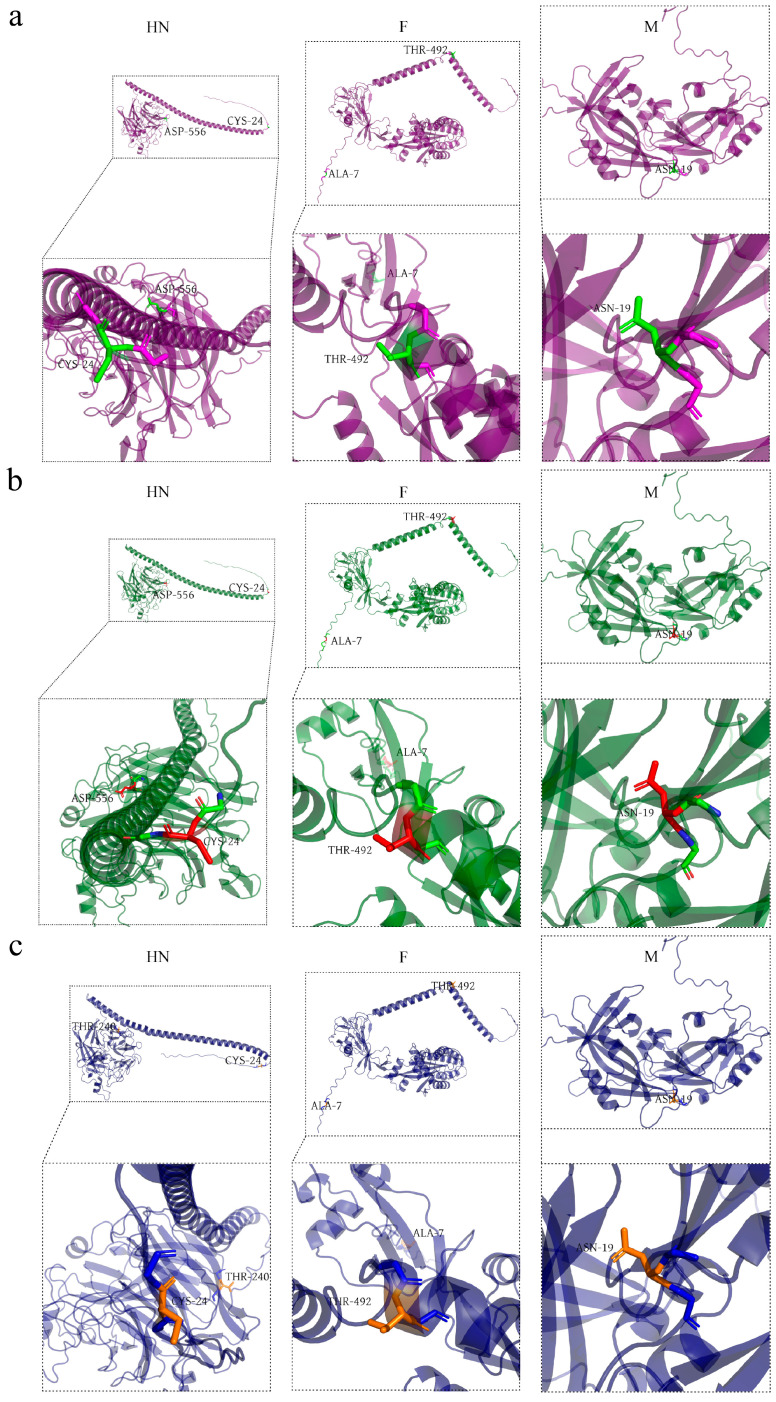
Protein mutation analysis of BPIV-3C isolates. The different colors of the proteins represent different isolates. (**a**) The cartoon schemes of HN, F, and M proteins of the Hubei-01 strain. Mutated amino acid sites are green. (**b**) The cartoon schemes of HN, F, and M proteins of the Hubei-02 strain. Mutated amino acid sites are red. (**c**) The cartoon schemes of HN, F, and M proteins of the Hubei-03 strain. Mutated amino acid sites are orange.

The isolates’ amino acids of N and P proteins were highly conserved, and no mutations were observed. The amino acid variations in different proteins of isolated strains can be found in Supplementary Table S6. All isolated strains’ M proteins displayed a common amino acid mutation site at ASN-19. The L protein of strains had two identical mutation sites, ASN-698 and VAL-1774 (protein too large to predict), while the F protein had two, ALA-7 and THR-492. In the HN protein region, Hubei-01, Hubei-02, and Hubei-03 had two amino acid mutation sites, one of which was a common mutation site of CYS-24, and the other mutated amino acids were Asn240, Asn240, and Thr240, respectively (Figure 6).

### 3.4. Pathogenicity Detection of BPIV-3C

#### 3.4.1. Clinical Symptom Observation, Anatomical Pathology Examination, and Histopathological Changes

**Figure 7:**
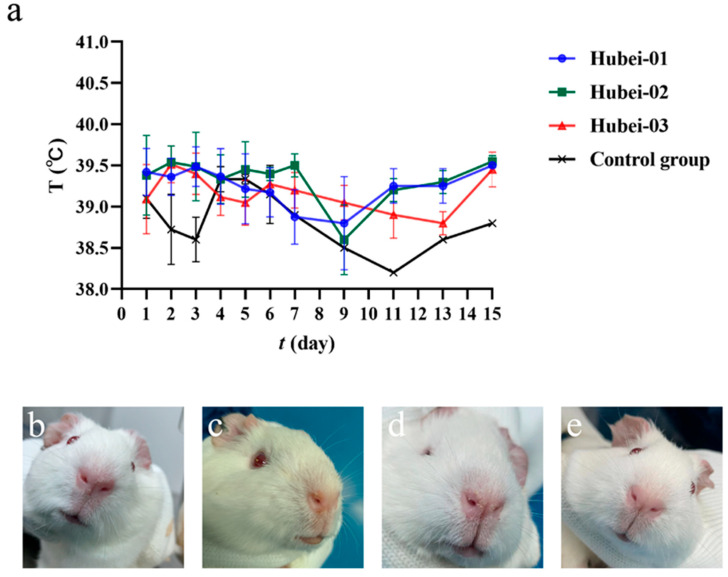
Clinical symptoms in guinea pigs post-infection. (**a**) Temperature changes in guinea pigs after infection. (**b**) Runny nose of guinea pig in Hubei-01 infection group. (**c**,**d**) are runny noses of guinea pigs in the Hubei-03 infection group. (**e**) Guinea pig in the control group.

Eight guinea pigs in the Hubei-01 infection group had a fever (39.6–39.9 °C) 1–5 days post-infection (Figure 7a). Two guinea pigs were depressed 11 days post-infection, and one had a runny nose on day 15 post-infection (Figure 7b).

Eight guinea pigs in the Hubei-02 infection group had a fever (39.6–40.4 °C) 1–7 days post-infection (Figure 7a), and two guinea pigs were depressed on 7–11 days post-infection.

Seven guinea pigs in the Hubei-03 infection group had a fever (39.6–39.9 °C) 1–3 days post-infection (Figure 7a). One guinea pig became depressed on day 2 post-infection and persisted until necropsy on day 7 post-infection. Additionally, 2 guinea pigs showed signs of depression and runny nose on day 7 post-infection, which persisted until necropsy on day 15 post-infection (Figure 7c,d).

The control group of guinea pigs maintained a normal body temperature (37.8–39.5 °C) throughout the infection period, with no clinical symptoms (Figure 7a,e).

**Figure 8:**
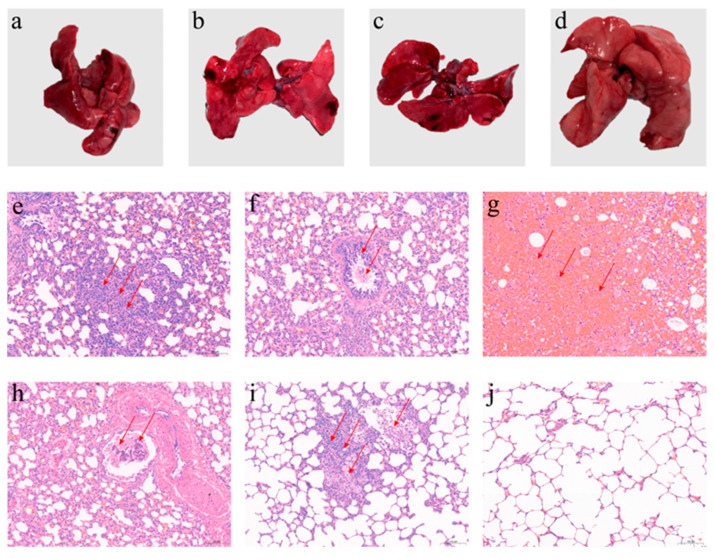
Histopathologic changes in the lungs of guinea pigs post-infection. Pathological changes were marked with red arrows. (**a**–**c**) are congestion, stasis, and consolidation in the lungs of guinea pigs from the Hubei-01, Hubei-02, and Hubei-03 infection groups, respectively. (**d**) Normal lung tissue in the control group of guinea pigs. (**e**) Proliferation of alveolar epithelial cells (200×). (**f**) Shedding and proliferation of bronchiolar epithelial cells (200×). (**g**) Severe hemorrhage in the alveoli (200×). (**h**) Necrosis of bronchiolar epithelial cells (200×). (**i**) Infiltration of lymphocytes and macrophages (200×). (**j**) Lung tissue section from the control group guinea pigs (200×).

In Groups 1–3 infected BPIV-3, from 5 days post-infection, the lungs showed obvious congestion, hemorrhage, atrophy, and localized consolidation, which were most severe 15 days post-infection (Figure 8a–c). Histopathological results showed interstitial pneumonia in all infected animals, presented as proliferative changes in the alveolar epithelial cells, infiltration of inflammatory cells mainly consisting of macrophages and lymphocytes within the alveolar interstitium, dilation, and congestion of the interstitial capillaries, interstitial hemorrhage and stasis, concurrent necrosis and shedding of the epithelial cells of the bronchioles (Figure 8e−i).

**Figure 9:**
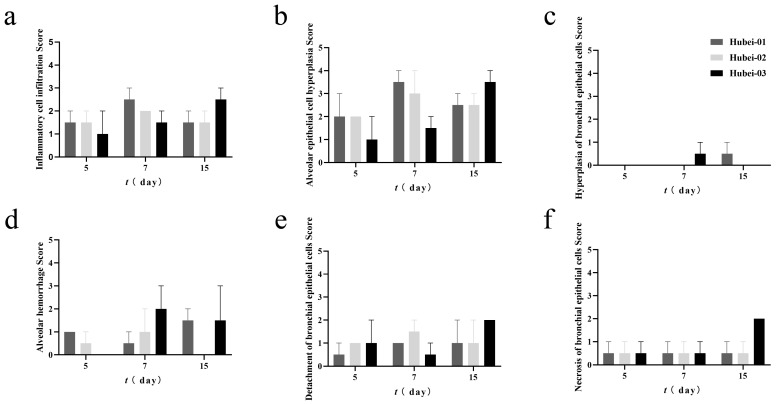
Scoring of pathological changes in guinea pig lung tissue. There was no significant difference by two-way ANOVA. (**a**) Inflammatory cell infiltration. (**b**) Alveolar epithelial cell hyperplasia. (**c**) Hyperplasia of bronchial epithelial cells. (**d**) Alveolar hemorrhage. (**e**) Detachment of bronchial epithelial cells Score. (**f**) Necrosis of bronchial epithelial cells.

Then, the lung pathological changes were scored. The results indicated that with the progression of the infection duration, the overall lung pathological changes in the Hubei-03 infection group showed a trend of gradual worsening. Lesions such as proliferation of alveolar epithelial cells, infiltration of inflammatory cells, necrosis, and shedding of bronchiolar epithelial cells were most severe on day 15 post-infection, with average scores reaching 3.5, 2.5, 2.0, and 2.0, respectively. Hemorrhage in the alveoli was noted to start appearing on day 7 post-infection, showing its most severe manifestation with an average score of 2.0. Meanwhile, the proliferation of bronchiolar epithelial cells was observed only on day 7 post-infection, albeit to a lesser extent, with an average score of 0.5 (Figure 9). In the Hubei-01 infection group, specific lung pathological changes were most severe on day 7 post-infection, such as the proliferation of alveolar epithelial cells and infiltration of inflammatory cells, with average scores of 3.5 and 2.5, respectively. The necrosis lesions and shedding of bronchiolar epithelial cells had consistent average scores of 0.5 and 1.0 on both day 7 and day 15 post-infection. Proliferation of bronchiolar epithelial cells was only observed on day 15 post-infection, with an average score of 0.5 (Figure 9). Like the Hubei-01 group, most of the lung tissue pathological changes in the Hubei-02 infected group were most severe on day 7 post-infection, such as the proliferation of alveolar epithelial cells, inflammatory cell infiltration, alveolar hemorrhage, and bronchiolar epithelial cell shedding, with average scores of 3.0, 2.0, 1.0, and 1.5 respectively. The bronchiolar epithelial cell necrosis lesion had consistent average scores of 0.5 on days 5, 7, and 15 post-infection. Throughout the infection period, no lesions related to the proliferation of bronchiolar epithelial cells were observed (Figure 9). Among the three infection groups, the Hubei-03 group had higher overall lung pathological change scores.

No pathological changes were detected in the control groups (Figure 8d,j).

#### 3.4.2. Detoxification Duration and Organ Viral Load

On 1–3 days post-infection, viruses were successfully isolated from nasal swabs of all guinea pigs in the infection groups. qPCR results showed that nasal detoxification of guinea pigs in the Hubei-01 and Hubei-03 infection groups lasted until the 5th day after infection, while the Hubei-02 infected group lasted until the 7th day. No virus was isolated and detected from nasal swabs of the control (Supplementary Table S7).

**Figure 10:**
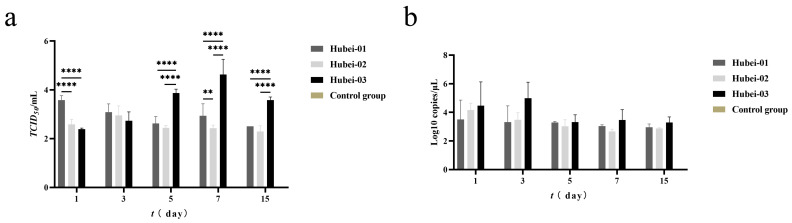
The viral load in guinea pig lung tissue post-infection. (**a**) Viral titer in the lungs post-infection. Tested by two-way ANOVA. ** represents *p* < 0.01. **** represents *p* < 0.0001. (**b**) Viral copy numbers in the lungs post-infection.

The viral load in the lungs of guinea pigs was then assessed. On day 1 post-infection, the Hubei-01-infected group showed a significantly higher viral load than the other infection groups (*p* < 0.001); the TCID_50_ was as high as 10^3.58^/mL. However, the Hubei-03-infected group revealed a significantly higher viral load 5 days post-infection as compared to the other two infected groups, as high as 10^3.87^/mL on day 5, TCID_50_ of 10^4.63^/mL on day 7, and 10^3.58^/mL on day 15 (*p* < 0.0001) (Figure 10a). In addition, qPCR showed that the Hubei-03-infected group possessed the highest viral copy number throughout the observation period (Figure 10b).

No viral titers and copy numbers were detected in the control guinea pigs (Figure 10a,b).

#### 3.4.3. Neutralizing, Specific, and Hemagglutination Inhibiting Antibodies

Neutralization antibodies were not detected until day 7 post-infection. On day 15 post-infection, the antibody potency reached 1:6 in all infected groups (Supplementary Table S8). There was no significant difference among all infected groups. No neutralizing antibodies were detected in the control group.

**Figure 11:**
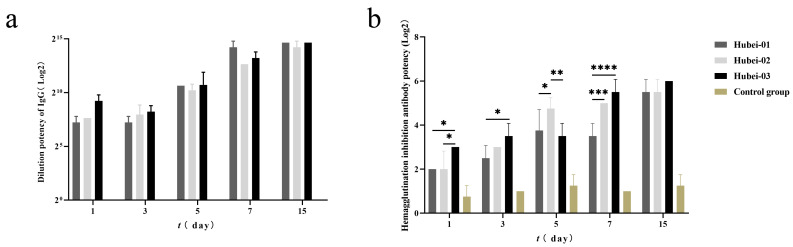
Specific antibody and hemagglutination inhibition antibody titers in guinea pig serum post-infection. (**a**) Specific antibody titers in guinea pig serum. (**b**) Hemagglutination inhibition antibody titers in guinea pig serum. Tested by two-way ANOVA. * represents *p* < 0.05. ** represents *p* < 0.01. *** represents *p* < 0.001. **** represents *p* < 0.0001.

The same trend occurred in the specific IgG antibody detection. The antibody titers of all infected groups increased as the days went by. By day 15 post-infection, the serum-specific IgG antibody titers reached their peaks, with dilution titers of 1:25,600, 1:25,600, and 1:19,200, respectively (Figure 11a). Throughout the whole period, no specific IgG antibodies were detected in the control group.

Following that, to detect more hemagglutination inhibitory antibodies, we calculated the hemagglutination titers of various isolates. The hemagglutination titers of Hubei-03 (2^9^) showed a much higher titer than the Hubei-01 and Hubei-02 strains (both were 2^1^) (Supplementary Figure S1). Therefore, we prepared a four-unit antigen using the Hubei-03 virus strain for subsequent hemagglutination inhibition tests. The results (Figure 11b) showed that the hemagglutination inhibition antibody titers in the infected group gradually increased with the progression of the infection duration. Compared to the other infection groups, the Hubei-03 infection group consistently had the highest antibody levels (except on day 5 post-infection), reaching a titer of 26 on day 15 post-infection.

## 4. Discussion

BPIV-3 is the most important viral respiratory pathogen in calves and adult cattle [4]. To our knowledge, however, very little is known about the outbreak in China. Here, we reported a 13.16% (95% CI: 4.41–28.09) (5/38) BPIV-3 herd-level and 4.64% (95% CI: 2.56–7.66) (14/302) individual-level proportion in cattle, indicating that BPIV-3 commonly exists in cattle farms with BRDC outbreaks in China, which is consistent with a previous limited study [17]. The predominant genotype of BPIV-3 in China is C [5,14,18], which also aligned with our results.

### 4.1. BPIV-3C Is the Predominant Genotype in China, with a Low Genetic Diversity

Understanding the phylogeny of BPIV-3 is an important basis for clarifying its evolution, sources of transmission, and molecular epidemiology, and can improve our ability to analyze and predict disease re-emergence [1,19]. This study found that genotype C strains of BPIV-3 had a lower genetic diversity. The identity analysis showed that from 2008 to 2023, genotype C strains isolated from different countries had a high identity, as high as 97%, and were located in an independent large lineage with low differentiation. For genotype A and B, the identity was significantly lower than C, and the strains gave rise to multiple sub-branches, as previously reported [14,20].

As HN, P, N, and M genes of BPIV-3 were regarded as suitable genes for phylogenetic analysis and genotyping [20,21,22], we compared HN, P, N, and M phylogenetic analysis and genotyping with the whole-genome sequence. We found that the HN, P, and N gene analysis results were much more accurate than for the M gene because M proteins are highly conserved.

### 4.2. Geographical Location and Trade May Influence the Spread of BPIV-3

Geographical location is a risk factor for the virus’s transmission. After first being reported in China in 2008 [13], BPIV-3 genotype C gradually spread to neighboring countries [11,21]. Based on our study and the known BPIV-3 genomic sequences, the majority of genotype C strains were shown to have originated in China and its neighboring countries, a finding consistent with previous research. The expansion of global trade has facilitated the spread of the virus, as evidenced by the detection of genotype C strains in regions far from China, such as the USA and Turkey [23,24].

Similarly, the first reports of genotype A strains in China have also illuminated the impact of international trade on the spread of the virus [25]. According to this study’s identity and phylogenetic analysis, the strains found in Hubei were most closely linked to the NX49 strain found in Ningxia. Despite their lack of proximity, Hubei and Ningxia are often involved in the trade of beef and raising cattle, which increases the possibility that the prevalence of isolates in Hubei is directly linked to the transportation of livestock and related goods.

### 4.3. The Hubei-02 Strain Was Involved in the Genetic Evolution of the NX4 Strain

Homologous recombination plays a key role in the evolution of negative-stranded RNA viruses [26], promoting molecular diversity in viruses and introducing new biological traits [27]. There is currently little information available on the homologous recombination of BPIV-3 strains. Recombination events were only found in BPIV-3 strains originating from pigs, although some researchers had examined the entire genomes of 25 BPIV-3 strains from various sources [1]. Our investigation demonstrated for the first time the homologous recombination events in BPIV-3 strains of bovine origin with parental strains from different locations, indicating the trans-regional nature of genetic recombination. Hubei-02 strain in this study was the parental strain in the recombination event of NX4 strain, suggesting that Hubei-02 was involved in the genetic evolution of the NX4 strain. These findings will provide new evidence for the role of homologous recombination in the evolution of BPIV-3.

### 4.4. Mutations in Protein Amino Acids May Have Altered the Function of the Isolates

The HN and F proteins of paramyxoviruses are indispensable in viral infection. HN protein acts as a multifunctional glycoprotein recognizing salivary acid containing receptors on the cell surface and enhancing the fusion activity of F proteins; it also acts as a neuraminidase or salivary acid enzyme, scavenging salivary acid from progeny viral particles and preventing viral self-accumulation [28]. The 156–174, 191–203, 515–527, and 547–556 aa regions of HN protein are sialic acid second binding sites, and mutations in the amino acids of these regions affect the neuraminidase activity, receptor-binding capacity, and cell fusion-promoting ability of Newcastle disease virus (NDV) [28,29]. Although BPIV-3 and NDV belong to the family Paramyxoviridae, there is no evidence that mutations in the HN protein’s second sialic acid binding site affect the neuraminidase activity of BPIV-3. In this study, the Asp556 residue of the HN protein’s second salivary acid binding site was mutated in Hubei-01 and Hubei-02 isolates. Notably, subsequent hemagglutination tests showed that hemagglutination titers of the Hubei-01 and Hubei-02 isolates were significantly lower than that of the Hubei-03 isolate, suggesting that the mutation of the Asp556 residue was very likely to reduce the HA activity of the Hubei-01 and Hubei-02 strains, thus reducing their ability to adsorb red blood cells. F protein is a homotrimer that mediates the fusion of the viral envelope with the host cell membrane and viral entry [30,31]. The 1–18 aa region of F protein is a signal peptide structural domain associated with the regulation of viral fusion functions [30,32], indicating that the mutation at the Ala7 amino acid site of the F protein in the Hubei-01, Hubei-02, and Hubei-03 isolates might have affected the virus’s fusion function. In addition, other amino acid mutation sites were observed in the HN, F, and other proteins of the Hubei-01, Hubei-02, and Hubei-03 isolates in this study, but the roles and effects of these sites on the viruses are unknown.

### 4.5. The Hubei-03 Strain Had the Highest Virulence among All Isolates

There is currently no commercial vaccine for BPIV-3 in China. To screen for effective vaccine candidates, we further evaluated the pathogenicity of the isolates against guinea pigs. The results showed that all guinea pigs developed significant respiratory symptoms and pathological lung changes after being intranasally inoculated with the isolates. Our findings differed from previous investigations [18,33,34,35,36], which may be related to the animal species and the virulence of the strains. Among three infection groups, guinea pigs inoculated with Hubei-03 had the most obvious clinical symptoms, the most obvious pathological changes, and the highest viral load in the lungs, which indicated that the pathogenicity of the Hubei-03 strain had the highest virulence among all the isolated viruses. Further studies are needed to confirm whether these isolates’ pathogenicity differences are related to protein variants.

## 5. Conclusions

Six BPIV-3 strains were isolated from 14 positive cattle. BPIV-3C is the primary genotype in China, having low genetic diversity, and the prevalence of the isolates in this study may be associated with the transport of cattle and related goods. Genetic recombination can also occur in bovine-origin BPIV-3; protein amino acid mutations may affect the isolates’ function. The most pathogenic strain was Hubei-03, which could be exploited as a vaccine candidate.

## Figures and Tables

**Table 1:** Pathological slice scoring criteria.

Scoring	Kinds	Clarification	Reference
0	Within normal limits	In the research, factors like the animals’ age, gender, and strain were considered, and the tissue was deemed normal. Any changes outside these conditions were viewed as abnormal.	[16]
1	Very mild	The changes that emerged were just beyond the normal range.	
2	Mild	The lesions were observed, but they had not yet become severe.	
3	Moderate	The lesions were obvious and likely to be more severe.	
4	Severe	The lesions were extremely severe (the lesions had occupied the entire tissue or organ).	

## Data Availability

The sequences generated in this study have been submitted to GenBank (ncbi.nlm.nih.gov) under accession numbers OR855359-OR855361.

## Supplementary Materials

The following supporting information can be downloaded at: https://www.mdpi.com/article/10.3390/v16030402/s1, Table S1: Samples distribution; Table S2: List of primers and probe used in this study; Table S3: Reference strains used for the phylogenetic analysis of BPIV-3 in this study; Table S4: Genomic information of BPIV-3 isolates; Table S5: RDP4 program to analyze genomic recombination events of BPIV-3 strains; Table S6: Unique amino acid sequences of different proteins of BPIV-3 isolates; Table S7: Results of virus isolation and qPCR testing from guinea pig nasal swabs post-infection; Table S8: Neutralizing antibody titers in the serum of the infected group; Figure S1: Hemagglutination test.
