# Supplementary material for: Prevalence, Molecular Characteristics and Virulence Identification of Bovine Parainfluenza Virus Type 3 in China
Source: Viruses. 2024 Mar 5;16(3):402. doi: 10.3390/v16030402 (PMC10974836; doi:10.3390/v16030402)
Supplement: Supplementary file 1 [file viruses-16-00402-s001.zip › viruses-2888393-supplementary materials.pdf]

Supplementary materials

Table S1. Samples distribution

Provinces/ Autonomous regions	Farm No.	Cattle No.
Hubei	16	149
Anhui	2	8
Guangxi	2	20
Guizhou	1	12
Hebei	2	8
Henan	1	30
Gansu	9	47
Hunan	2	11
Ningxia	1	10
Zhejiang	2	7
Total	38	302

Table S2. List of primers and probe used in this study

	Sequence	Product size (bp)
Primer 1 (BPIV-3)	F: GAGAAAGACCCAGGAAGACAGA<br>R: ACACCCATCGCATAACTCCAGA	704
Primer 2 (Genotype A)	F: CATCCATAGTTCCTTATGCATG<br>R: TTGGGTCGCTCTGTTTCC	153
Primer 3 (Genotype C)	F: ACAGTATGTAACAGGACGGTCC<br>R: GCCTCTTGTGTAATCCCCAA	126
Probe	P: HEX-TGCCACTGCTTGACCTAGTTGGAAC-BHQ1	

Table S3. Reference strains used for the phylogenetic analysis of BPIV-3 in this study

Strains	Hosts	Country of origin	Genotype	GenBank accession number
NX49	Bovine	China	C	KT071671
XJ20055-3	Bovine	China	C	OM632676
HB2	Bovine	China	C	OP718793
LT2	Yak	China	C	OM782291
SC20	Yak	China	C	OM621819
NM2	Bovine	China	C	OP718792
NX4	Bovine	China	C	OP718794
LT1	Yak	China	C	OM782290
JL6	Bovine	China	C	OP718795
SC39	Bovine	China	C	OP718796
XJA13	Bovine	China	C	KU198929
SD2020	Bovine	China	C	MZ359081
SX6	Bovine	China	C	OP718797
Turkey_S1	Bovine	Turkey	C	MH357343
SD0835	Bovine	China	C	HQ530153
TVMDL16	Bovine	USA	C	KJ647285
FK/D56/13	Bovine	Japan	C	LC040886
12Q061	Bovine	South Korea	C	JX969001
HS9	Bovine	Japan	C	LC000638
TVMDL20	Bovine	USA	C	KJ647287
Tottori3c	Bovine	Japan	C	LC200422
D1189.8	Camelus dromedarius	United Arab Emirates	-	MW504259
D1189.2	Camelus dromedarius	United Arab Emirates	-	MW504257
D1189.4	Camelus dromedarius	United Arab Emirates	-	MW504258
Shipping Fever	Rhesus monkey	USA	A	AF178655
Kansas/15626/84	Rhesus monkey	USA	A	AF178654
NC_002161	Rhesus monkey	USA	A	-

TVMDL60	Bovine	USA	A	KJ647289
3/Egypt/2014	Bovine	Egypt	A	KP757872
NM09	Bovine	China	A	JQ063064
TtPIV-1	Dolphin	USA	B	KP764763
TVMDL17	Bovine	USA	B	KJ647286
TVMDL15	Bovine	USA	B	KJ647284
XJ21032-1	Bovine	China	B	ON081628
Q5592	Bovine	Australia	B	EU277658
BN-CE	Bovine	Japan	A	AB770485
910N	Bovine	Japan	A	D84095.1
BPIV3 BJ	Bovine	China	A	MH552577
BN-1	Bovine	Japan	A	AB770484
TVMDL24	Bovine	USA	A	KJ647288

Table S4. Genomic information of BPIV-3 isolates

strains	Abbreviation	Genotype	Genome length	GC (%)	Regions (nt)					
					N	P	M	F	HN	L
BPIV-3C/Bovine/Hubei/1/2022	Hubei-01	C	15465	36.68	105-1652	1778-3580	3741-4796	5090-6712	6812-8530	8652-15353
BPIV-3C/Bovine/Hubei/2/2022	Hubei-02	C	15460	36.67	104-1651	1777-3579	3740-4795	5089-6711	6811-8529	8651-15352
BPIV-3C/Bovine/Hubei/3/2022	Hubei-03	C	15462	36.69	104-1651	1777-3579	3740-4795	5089-6711	6811-8529	8651-15352

Table S5. RDP4 program to analyze genomic recombination events of BPIV-3 strains

Recombination		Event 1	Event 2	Event 3
strains		NX4	JL6	12Q061
Beginning breakpoint without gaps (nt 99% CI)		3530 (2462-4024)	11427 (11324-410)	10378 (6627-8103)
Ending breakpoint without gaps (nt 99% CI)		5264 (4982-5794)	2768 (2217-2859)	11161 (8122-9445)
Major parent		LT1	SD0835	Tottori3c
Minor parent		BPIV-3C Hubei-02	NX4	NM2
Methods	RDP	/	2.120×10^{-05} (*P*-Val)	8.925×10^{-03} (*P*-Val)
	GENECONV	/	1.138×10^{-11} (*P*-Val)	6.866×10^{-08} (*P*-Val)
	Chimaera	2.317×10^{-02} (*P*-Val)	3.506×10^{-03} (*P*-Val)	/
	MaxChi	2.338×10^{-03} (*P*-Val)	1.026×10^{-02} (*P*-Val)	/
	BootScan	/	2.146×10^{-05} (*P*-Val)	7.720×10^{-03} (*P*-Val)
	SiScan	/	/	2.979×10^{-03} (*P*-Val)
	3Seq	4.142×10^{-06} (*P*-Val)	1.277×10^{-05} (*P*-Val)	/

Table S6. Unique amino acid sequences of different proteins of BPIV-3 isolates

strains	Location of amino acid variation for different proteins（aa）							
	M	L		F		HN		
	19	698	1774	7	492	24	240	556
Consensus	Ser	Asp	IIe	Thr	IIe	Arg	Asn	Asn
Hubei-01	Asn	Asn	Val	Ala	Thr	Cys	Asn	Asp
Hubei-02	Asn	Asn	Val	Ala	Thr	Cys	Asn	Asp
Hubei-03	Asn	Asn	Val	Ala	Thr	Cys	Thr	Asn

Table S7. Results of virus isolation and qPCR testing from guinea pig nasal swabs post-infection

**Grouping**	**DPI** [a]	**Cultured in cells**	**qPCR**
Hubei-01	1	（10[1]/10[2]）	（10[3]/10[4]）
	3	（8/8）	（8/8）
	5	（0/6）	（4/6）
	7	（0/4）	（0/4）
Hubei-02	1	（10/10）	（10/10）
	3	（8/8）	（8/8）
	5	（0/6）	（3/6）
	7	（0/4）	（2/4）
Hubei-03	1	（10/10）	（10/10）
	3	（8/8）	（8/8）
	5	（0/6）	（3/6）
	7	（0/4）	（0/4）

[a] Days post-infection in guinea pigs.
[1] The number of guinea pig nasal swabs that can cause cytopathic effect after being inoculated with cells.
[2, 4] The total number of guinea pig nasal swabs collected after infection, one nasal swab per guinea pig. As the number of infected days increased, the number of guinea pigs gradually decreased due to dissection.
[3] The number of guinea pig nasal swabs that tested positive for qPCR.

Table S8. Neutralizing antibody titers in the serum of the infected group

**DPI** [a]	**Mean antibody titer of infected groups**		
	Hubei-01	Hubei-02	Hubei-03
7	3	3	3
15	6	6	6

[a] Days post-infection in guinea pigs.

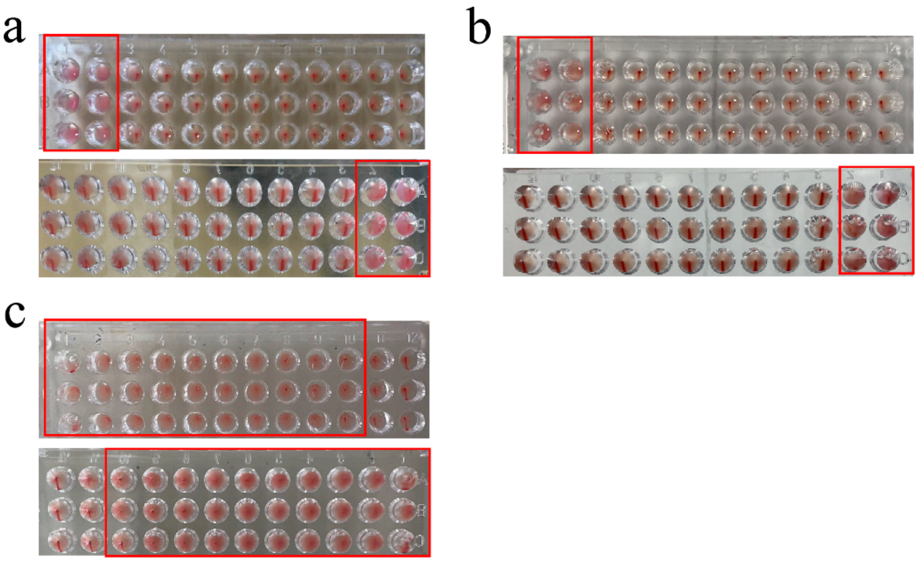

Figure S1. Hemagglutination test. (**a**) Hemagglutination potency of BPIV-3C Hubei-01. (**b**) Hemagglutination potency of BPIV-3C Hubei-02. (**c**) Hemagglutination potency of BPIV-3C Hubei-03.
